# Mechanical regulation of signal transduction in angiogenesis

**DOI:** 10.3389/fcell.2022.933474

**Published:** 2022-08-19

**Authors:** Jennifer Flournoy, Shahad Ashkanani, Yun Chen

**Affiliations:** ^1^ Department of Mechanical Engineering, Johns Hopkins University, Baltimore, MD, United States; ^2^ Center for Cell Dynamics, Johns Hopkins University, Baltimore, MD, United States; ^3^ Institute for NanoBio Technology, Johns Hopkins University, Baltimore, MD, United States; ^4^ Department of Chemical and Biomolecular Engineering, Johns Hopkins University, Baltimore, MD, United States

**Keywords:** angiogenesis, ECM, stiffness, mechanotransduction, stretch, shear stress

## Abstract

Biophysical and biochemical cues work in concert to regulate angiogenesis. These cues guide angiogenesis during development and wound healing. Abnormal cues contribute to pathological angiogenesis during tumor progression. In this review, we summarize the known signaling pathways involved in mechanotransduction important to angiogenesis. We discuss how variation in the mechanical microenvironment, in terms of stiffness, ligand availability, and topography, can modulate the angiogenesis process. We also present an integrated view on how mechanical perturbations, such as stretching and fluid shearing, alter angiogenesis-related signal transduction acutely, leading to downstream gene expression. Tissue engineering-based approaches to study angiogenesis are reviewed too. Future directions to aid the efforts in unveiling the comprehensive picture of angiogenesis are proposed.

## Introduction

Angiogenesis, the growth of new blood vessels from existing vasculature, is a multi-step process. Angiogenesis begins with endothelial cells receiving a proangiogenic signal from a growth factor such as angiogenin (ANG), vascular endothelial growth factor (VEGF), platelet derived growth factor (PDGF), epidermal growth factor (EGF), fibroblast growth factor (FGF), transforming growth factor- *β* (TGF-β), and tumor necrosis factor *α* (TNF-α) (Zimta et al., 2019). Morphological changes to endothelial cells where the cells are thickened, and the amount of endoplasmic reticulum, ribosomes, and Golgi apparatus increase (Egginton et al., 2001). Pericytes detach from endothelial cells and endothelial cells release metalloproteases to degrade the extracellular matrix (Van Hove and Benoit, 2015). In response to VEGF cells develop tip cell or stalk cell phenotypes (Blanco and Gerhardt, 2013). Tip cells migrate into the extracellular matrix and stalk cells proliferate resulting in vascular tubes (Blanco and Gerhardt, 2013). Pericytes are recruited to the new vessel (Van Hove and Benoit, 2015). Capillary sprouts branch and link with other branches to form loops. Angiogenesis is of significant interest because it has a major role in embryonic development, cancer progression, and wound healing (Folkman, 1984). There are two types of angiogenesis: sprouting and non-sprouting (intussusception) (Risau, 1997). For this review, the focus will be on the initiation of sprouting angiogenesis.

During embryonic development, the primordial vascular system is formed by vasculogenesis (Risau and Flamme, 1995). Following the formation of the primary vascular plexus new capillaries are formed by sprouting angiogenesis or intussusception (Risau, 1997). The vascular system develops to meet the oxygen and nutritional requirements of the embryo (Breier, 2000). Angiogenesis also occurs during wound healing. Wound healing consists of three stages: inflammation, new tissue formation, and remodeling (Gurtner et al., 2008). During the new tissue formation stage capillary sprouts replace the fibrin matrix (Gurtner et al., 2008). The sprouting of new vessels into the wound provides oxygen and nutrients to the wound site as well as a means for leukocytes to reach the wound (Li et al., 2003). Angiogenesis also plays a critical role in cancer progression. It has been known that angiogenesis contributes to cancer progression for over a century. Blood vessels were identified in malignant growths over 100 years ago (Goldmann, 1908). Sustained angiogenesis is critical for cancer development (Hanahan and Weinberg, 2000). Without a blood supply to deliver oxygen and nutrients and remove waste, tumors cannot grow larger than a few millimeters (Hanahan and Weinberg, 2000). “Anti-angiogenesis” is a therapeutic strategy for cancer because blocking neovascularization limits tumor growth (Sherwood et al., 1971). However, some tumors gain the ability to induce and sustain angiogenesis (Hanahan and Weinberg, 2000). This is referred to as the “angiogenic switch.” Triggers for the “angiogenetic switch” include metabolic stress, immune/inflammatory response, and genetic mutations (Carmeliet and Jain, 2000). Unlike the highly orchestrated angiogenesis during embryonic development, tumor angiogenesis is a disorganized process. Tumor cells secrete VEGF to induce angiogenesis. Excessive VEGF impairs the vascular barrier function in tumor vasculature (Weis and Cheresh, 2005). Furthermore, tumor vasculatures often lack perivascular cells and contain tumor cells (Benjamin et al., 1999; McDonald and Foss, 2000).

We note that in addition to angiogenesis, blood vessels arise *via* vasculogenesis. Four steps, distinct from those of angiogenesis, take place in vasculogenesis. First, the mesoderm is formed. Next, blood island differentiation occurs within the mesoderm. Within the blood islands, the mesoderm cells differentiate into angioblasts and hemopoietic cells. Subsequently, the angioblasts differentiate into endothelial cells, the lumen is formed, and the basement membrane emerges. As the blood islands grow and endothelial cells migrate, blood islands become connected forming a primary capillary plexus (Risau and Flamme, 1995). After the vascular plexus is formed early in embryonic development, new capillary vessels are formed *via* angiogenesis (Risau, 1997). While vasculogenesis primarily occurs during embryonic development, postnatal instances of vasculogenesis are known to occur in ischemic and malignant tissues (Asahara and Kawamoto, 2004). Vasculogenesis also plays an important role in cancer (Begg et al., 2011). Following radiation therapy vasculogenesis influences tumor growth (Ahn and Brown, 2009). Vasculogenesis is the *de novo* formation of vessels from angioblasts, while angiogenesis is the formation of new blood vessels from existing blood vessels. However, angiogenesis is considered a more common mechanism facilitating the development of new blood vessels. Therefore, we will focus our review on angiogenesis.

While biochemical signaling has been extensively studied, how mechanics, biochemistry, and the microenvironment are integrated to regulate angiogenesis has yet to be comprehensively elucidated. Recently emerging evidence shows that mechanical cues are indispensable in a wide range of signal transduction, including the ones governing angiogenesis. We aim to highlight how the biochemical cascades resulting from crosstalk with mechanosignaling pathways that are initiated by sensing the extracellular matrix (ECM) stiffness, shear stress, and tension contribute to angiogenesis.

## Signaling pathways of angiogenesis

### Growth factors

**FIGURE 1:**
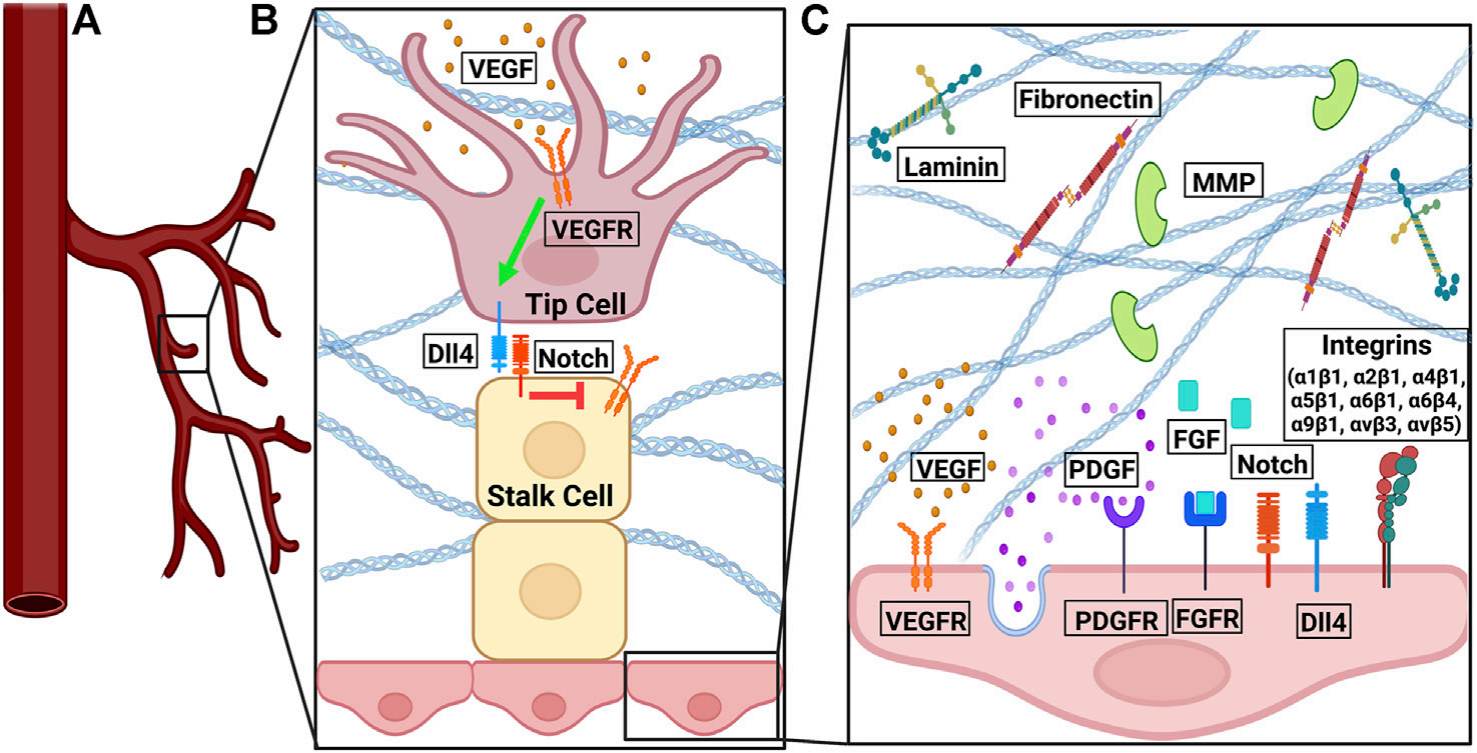
The signaling pathways involved in angiogenesis. **(A)** At the front of a sprouting vessel, **(B)** VEGF binds to VEGFR on tip cells. VEGFR activation induced the expression of Dll4. Dll4 on the tip cells binds to Notch on stalk cells. Notch signaling in stalk cells downregulates the expression of VEGFR2/3 in stalk cells. **(C)** VEGF-VEGFR and FGF-FGFR signaling promote pro-angiogenic signals, cell proliferation, and cell survival. PDGF-PDGFR binding leads to vessel sprouting and stabilization. Notch interacts with Dll4 in a feedback loop to regulate cell migration and proliferation. Fibronectin and laminin bind to integrins to promote endothelial cell proliferation, survival, migration, and tube formation. Various integrin isoforms promote angiogenesis through different overlapping pathways. MMPs degrade denatured collagen in the basement membrane.

Angiogenesis is regulated by a plethora of biomolecules belonging to overlapping pathways (Figure 1). Most prominent among them are growth factor signaling, ECM composition, and integrin signaling. Fibroblast growth factors (FGFs) facilitate migration and proliferation in angiogenesis by stimulating VEGF, mediating integrin levels, and activating proteolytic activity through tight binding to their respective receptors (FGFRs) (Otrock et al., 2007; Shoeibi et al., 2018). Vascular endothelial growth factor (VEGF) binds to tyrosine kinases on the cell surface, such as the VEGF receptor (VEGFR). Upon binding, VEGFR initiates pro-angiogenic signals, cell proliferation, migration, and survival (Abhinand et al., 2016). Platelet-derived growth factors (PDGFs) stimulate the proliferation of pericytes and smooth muscle cells, increase DNA synthesis and sprouting in endothelial cells *in vitro*, and stabilize vessels through binding to PDGF-beta receptors (Risau, 1997). Transforming growth factor- *β* (TGF-β) has both pro- and anti-angiogenic properties: at low doses, it upregulates pro-angiogenic factors (e.g., VEGF); at high doses, it promotes basement membrane formation and inhibits endothelial cell growth (Otrock et al., 2007; Shoeibi et al., 2018).

There are two phenotypes of cells: tip cells and stalk cells. Tip cells are at the leading front of endothelial sprouts during sprout formation. The cells within the stalk of the endothelial sprout are stalk cells. Tip cells can be identified *in vivo* or *in vitro* by CD34 (Sialomucin) expression (Siemerink et al., 2012). VEGFR2 is originally expressed on the surface of quiescent vessels (Blanco and Gerhardt, 2013). VEGF-A binds to VEGFR2. Upon binding to VEGF-A, the quiescent cell becomes a tip cell and migrates along the VEGF-A gradient by filopodia (Gerhardt et al., 2003; Suchting et al., 2007). The differential expression of VEGFR on stalk and tip calls originates from the binding of VEGF-A to VEGFR2 on tip cells, which leads to increased expression of DII4. DII4 binds to the Notch ligand on stalk cells. Notch signaling in stalk cells decreased the expression of VEGFR2 (Blanco and Gerhardt, 2013). Notch is a mechanosensitive protein activatable by shear stress (Mack et al., 2017; Loerakker et al., 2018; Stassen et al., 2021). In tip cells, VEGF activity upregulates the expression of Notch ligand Dll4, which in turn sends negative feedback through Notch signaling. Dll4 from tip cells binds Notch in the stalk cells, resulting in a decrease in VEGFR2/3 on the stalk cells (Blanco and Gerhardt, 2013). VEGF-Dll4-Notch signaling restricts tip cell formation, determines the ratio of tip to stalk cells, and the branching pattern of new vessels (Hellström et al., 2007). The feedback loop between VEGF and DII4- Notch pathways (Figure 1) manifests in an oscillating manner, shuffling tip and stalk cells during angiogenic sprouting (Jakobsson et al., 2010).

### Integrins and proteases

Another important regulator of angiogenesis is integrin-ECM interaction. Integrins are a family of mechanosensitive, transmembrane proteins facilitating cell adhesion and involved in cell migration, proliferation, and survival (Avraamides et al., 2008; Park et al., 2020). Since the 1980s it has been long understood that capillary tube formation is the result of a series of mechanochemical integrations, in which multiple cell-matrix and cell-cell adhesions are formed. Endothelial cells form tubes by accumulating adhesive matrix tendrils and applying tension *via* integrin to their attachment points (Ingber and Folkman, 1989). The composition of the ECM dictates the outside-in signaling initiated from integrin (Cheresh and Stupack, 2008). ECM proteins provide different cues to be sensed by various integrin isoforms. For example, laminin is required for tube formation of endothelial cells (Kubota et al., 1988; Grant et al., 1989; Rüdiger et al., 2020). Collagen IV promotes neovascular elongation and prevents vascular regression (Bonanno et al., 2000), while collagen I does not support endothelial tube formation (Deroanne et al., 2001; Rüdiger et al., 2020). Furthermore, fibronectin plays an important role in endothelial cell growth. It has been shown that fibronectin null mice die during embryogenesis and have deformed and defective vasculature (George et al., 1993). Inhibition of the fibronectin matrix inhibits vascular smooth muscle cell growth (Mercurius and Morla, 1998). Fibronectin is upregulated during wound healing (Clark et al., 1982). Fibronectin controls endothelial cell proliferation, enhances endothelial cell migration, and endothelial cell survival (Ingber, 1990; Kim et al., 2000).

Several integrin isoform dimers have known roles in angiogenesis: α1β1, α2β1, α4β1, α5β1, α6β1, α6β4, α9β1, αvβ3, and αvβ5 (Avraamides et al., 2008). Among them, αvβ3, which binds to ECM proteins vitronectin, fibronectin, fibrinogen, and osteopontin (Avraamides et al., 2008), serves as a marker for angiogenic vascular tissue (Brooks et al., 1994a), and its blockade prevents neovascularization (Brooks et al., 1994a). Induction of angiogenesis by FGF and tumor necrosis factor *α* (TNF-α) promotes integrin αvβ3 expression. Integrin αvβ3 antagonists stall tumor progression by inducing apoptosis in angiogenic vascular cells but preexisting vasculature is unaffected (Brooks et al., 1994b). Different integrin isoforms promote angiogenesis through overlapping but different pathways. This redundancy imposes challenges in blocking angiogenesis by targeting integrin in the tumor microenvironment. For example, αvβ5 is involved in angiogenesis *via* signaling downstream to FGF and TNF-α (Friedlander et al., 1995), and α5β1 *via* signaling downstream to FGF, TNF-α, and interleukin 8 (IL-8); but not VEGF (Kim et al., 2000; Plow et al., 2000).

In addition, cells remodel the ECM during angiogenesis. For example, regenerating endothelium exhibits a discontinuous or absent basement membrane (Ausprunk and Folkman, 1977). Matrix metalloproteinases (MMPs), proteolytic enzymes that degrade denatured collagen in the basement membrane (Otrock et al., 2007; Shoeibi et al., 2018) are an important regulator in this regard. MMP-9 null mice exhibit abnormal vascularization (Vu et al., 1998). An intact matrix is required for such ECM remodeling (Sottile and Hocking, 2002). Capillary basement membrane breakdown due to angiostatic steroid treatment and heparin results in capillary retraction, endothelial rounding, and associated capillary regression (Ingber et al., 1986).

## Surveying the microenvironment *via* mechanosensing

### Stiffness

Mechanical cues of the microenvironment, including ECM stiffness and the substrate geometry, also regulate angiogenesis. The endothelium-bearing tissue exhibit stiffness ranging from 2 kPa to 2 MPa (Dessalles et al., 2021). ECM compositions and organizations govern the issue stiffness. Therefore, ECM has been fabricated with the stiffness in this range to represent *in vivo* conditions, and to be tested *in vitro* about the stiffness effect on angiogenesis. ECM stiffness is an important regulator of cell behavior and lineage (Engler et al., 2006; Wells, 2008). Dysregulation of tissue stiffness is implicated in many different disease such as cancer and hypertension (Zanotelli and Reinhart-King, 2018; Cai et al., 2021). Cells sense and respond to ECM stiffness using integrin molecules (Discher et al., 2005; Kechagia et al., 2019). ECM binding results in a conformational change of integrin from an inactive to an active state (Askari et al., 2009). Focal adhesion kinase (FAK), talin, vinculin, and other adaptor proteins are recruited to integrins (Serrels and Frame, 2012; Atherton et al., 2016). Talin and vinculin link integrins to F-actin, allowing mechanical coupling between the actomyosin network and the ECM, where traction forces by actin contraction can be transmitted from inside of the cell outward to ECM (Kechagia et al., 2019). On stiffer substrates, there is higher force transmission through integrins (Kechagia et al., 2019).

**FIGURE 2:**
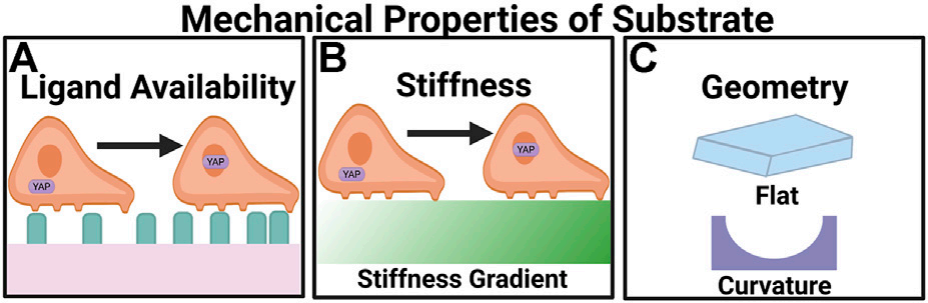
Angiogenesis is regulated by the mechanical properties of the microenvironment. **(A)** Ligand availability regulates the migratory behavior of cells through haptotaxis. In high ligand densities YAP translocates to the nucleus. **(B)** Greater stiffness upregulates the expression of pro-angiogenic factors. On stiff substrates YAP undergoes nuclear localization. **(C)** Appropriate curvatures of the substrates can promote angiogenesis.

Depending on ECM stiffness, the mechanical coupling between the actomyosin network and the ECM results in varying degrees of mechanosignaling, and the subsequent differential morphologies of endothelial cells (Figure 2). On soft substrates (∼0.2 kPa) endothelial cells are round, while on relatively stiff substrates (∼3 kPa), endothelial cells spread out (Yeung et al., 2005). Moreover, in 3D ECM endothelial cells exhibit durotaxis, where cells migrate along a rigidity gradient from low to high (Lo et al., 2000), and gather in the stiffest region (Joaquin et al., 2016). Proliferation is also affected by stiffness. For example, it was observed that phosphorylation increases extracellular signal-regulated protein kinase (ERK 1/2), an enzyme involved in cell division regulation, in cells subjected to stiff substrates (10 kPa) compared to the control (1 kPa) (LaValley et al., 2017). In addition, stiff substrates induce pro-angiogenesis gene expression. Stiff substrates increase the expression of genes encoding angiogenesis-related growth factors, such as *VEGFA*, *VEGFB,* hypoxia-inducible factor-1- *a* (HIFα), TGF-β, and epidermal growth factor (EGF) (Zhao et al., 2018). On stiff substrate, the actomyosin contractility is higher and promotes nuclear translocation of the transcription factor of YAP (Das et al., 2016), upregulating the expression of pro-angiogenesis genes. Ingber and colleagues showed that *VEGFR2* mRNA and protein expression were upregulated in human microvascular endothelial cells cultured on fibronectin polyacrylamide gels with a Young’s modulus of 4 kPa compared to cells cultured on 150-Pa gels (Mammoto et al., 2009). This trend is confirmed *in vivo*. Using a Matrigel implant assay it was determined that there is an optimal ECM stiffness (0.8 kPa) for angiogenesis *in vivo* (Mammoto et al., 2009). Consequently, endothelial cells are more proliferative on stiff ECM. Overall, these findings at the single-cell scale suggest that mechanosignaling events originating from ECM translate to altered cytoskeleton organization, enzymatic phosphorylation, and gene transcription/translation in the context of angiogenesis.

At the multi-cell scale, evidence abounds that angiogenesis is a highly mechanosensitive process. Endothelial sprouting is initiated by tip cells. Stiff ECM promotes translocation of Yes-associated protein (YAP) through Rho GTPases and tension in the actomyosin cytoskeleton (Dupont et al., 2011). Actomyosin tension is modulated by the phosphorylation of focal adhesion kinase (FAK) and paxillin, which also results in YAP translocation to the nucleus and VE-Cadherin degradation (Guo et al., 2022). When VE-Cadherin is degraded the cell-cell junctions are weakened, which along with YAP activation promotes tip cell specification (Guo et al., 2022). In stiff ECMs, a larger fraction of myosin II is located on the actin cortex to guide endothelial branching (Fischer et al., 2009). It is also notable that the angiogenic sprouts in relatively stiff ECM invade deeper into ECM (Lee et al., 2013). ECM stiffness also plays an important role in angiogenesis during tumor progression, because ECM of the tumor microenvironment is often stiffer than normal tissues (Acerbi et al., 2015; Rice et al., 2017; Bauer et al., 2020). Greater angiogenic sprouting, invasion, and neovascular branching are commonly observed in tumor microenvironments. On the other hand, VE-Cadherin, responsible for the endothelial barrier function, is downregulated because of the increased ECM stiffness within the tumor (Bordeleau et al., 2017). With endothelial barrier function impaired, the newly formed vasculature is leaky. The leaky vasculature is inefficient in facilitating gas exchange, leading to hypoxia. Moreover, it provides a gateway for cancer cell intravasation, the first step of distant-organ metastasis.

The availability of ECM molecules dictates cell spreading and determines whether endothelial cells form tubes, undergo apoptosis, or differentiate (Dike et al., 1999), all of which are involved in angiogenesis (Figure 2). When endothelial cells are cultured in suspension the majority of the cells undergo apoptosis; however, when endothelial cells are cultured on different-sized fibronectin-coated adhesive islands, apoptosis declined as the island size was increased, suggesting geometric control of ECM availability regulates endothelial cell viability (Chen et al., 1997). Ligand availability also promotes the translocation of YAP. Under high ligand density YAP localized to the nucleus independent of substrate stiffness (Stanton et al., 2019). In another demonstration, Ingber and colleagues showed that endothelial cells cultured on 10 μm-wide strips form tubes with lumen, whereas cells on 30-μm wide ones do not (Dike et al., 1999). Additionally, cells on 10- μm lines exhibit lower focal adhesions (FAs) density and smaller adherens junctions compared to those on 50- and 100-μm strips (Lei et al., 2012). ECM patterns influence vessel morphogenesis.

### Curvature

Curvature exerts effects on cell migration. At the cellular level, Verbridge and colleagues demonstrated the effect of curvature using collagen hydrogels, the perimeter of which is either round or with sharp edges (Figure 2). Both round and sharp angles occur in blood vessels. Sharp edges can be observed *in vivo* at bifurcation points within vessels where an existing vessel branches into two (Williamson et al., 1980; Billaud et al., 2011; Moy et al., 2013; Corliss et al., 2019; Ishiyama et al., 2019). The results showed that endothelial cell invasion frequency is the highest in the structures with the highest curvature index of 0.16 and sharpness angle of 28°. Round and sharp hydrogels showed no significant difference in invasion frequency (Hosseini et al., 2015). When endothelial cells are cultured in a microfluidic channel, endothelial cells invade from the corners of the channel rather than the side of the channel (Verbridge et al., 2013). Similar observations were made in endothelial cells grown in a curved channel, where sprouting is 1.24 fold-higher in the curved region compared to flat surfaces (Song et al., 2018). Distinctly differential transcriptional profiles are detected in vessels formed on a curved substrate by bulk RNA-seq, where genes promoting vascular growth are upregulated (Mandrycky et al., 2020). Interestingly, scRNA-seq showed there is a greater degree of overlap in gene expression between vessels formed on flat and curved surfaces (Mandrycky et al., 2020).

The curvature at the subcellular length scale also influences angiogenesis. Myosin II controls endothelial branching by minimizing cell-surface curvature. A positive feedback regulation between myosin II and curvature stabilizes myosin II, which minimizes the curvature of the local cell surface. The feedback cycles lead to directionally biased branch initiation and retraction (Elliott et al., 2015). When culturing human adipose-derived mesenchymal stem cells (MSCs) on a cell insert with an average roughness of 4.17 μm, high-affinity β1 integrin levels and activated FAK/ERK are increased, and Rho-associated protein kinase (ROCK) pathway is activated at curved areas, in turn upregulating VEGF. This result demonstrates that sub-micron curvature can trigger pro-angiogenic signaling (Li et al., 2017).

## Mechanotransduction in response to external force and the implication in angiogenesis

### Stretch

**FIGURE 3:**
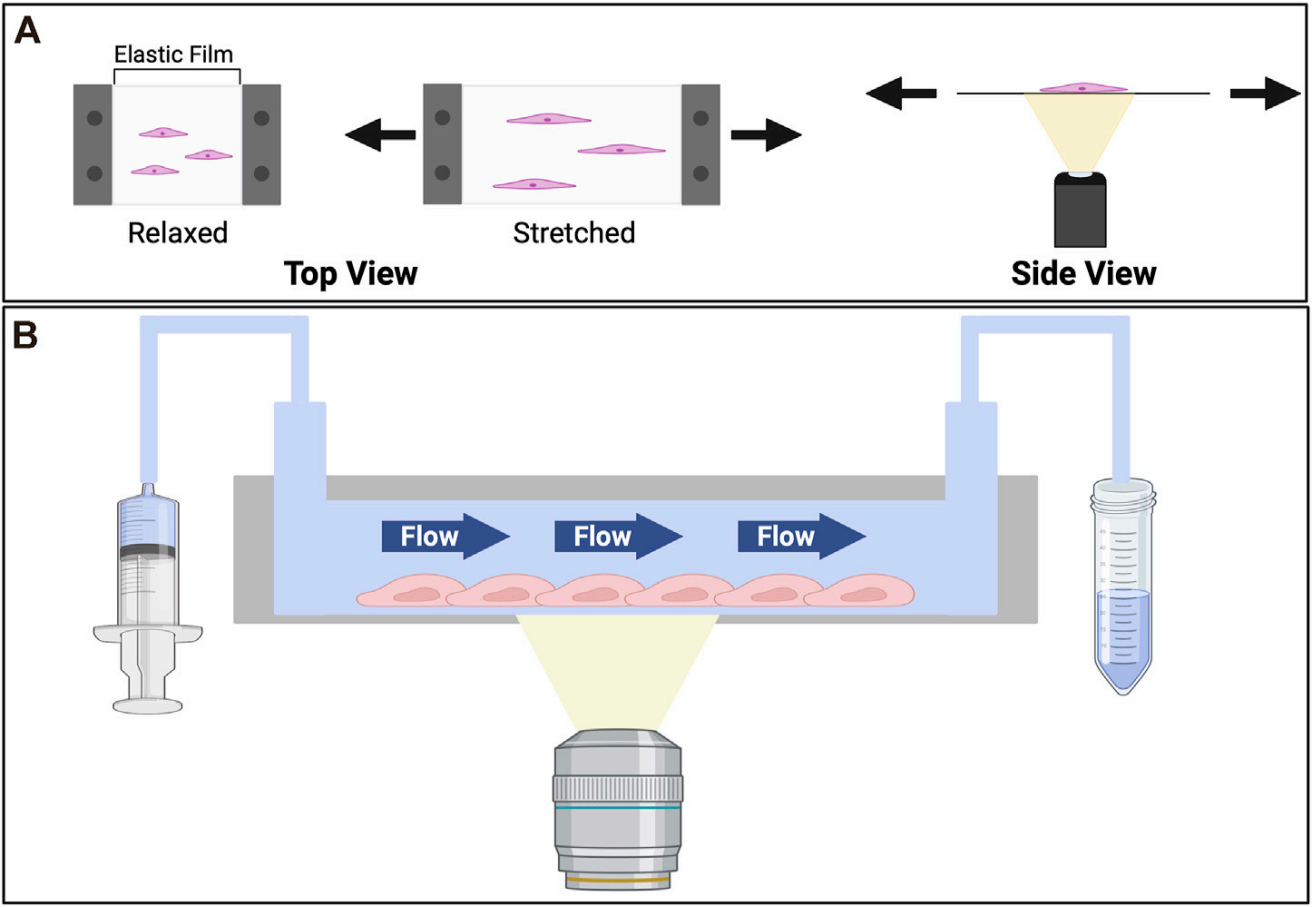
Devices for *in vitro* studies of effects imposed by the tensile stress (stretch) and shear stress (flow). **(A)** Cell stretcher. To observe the effects of stretch cells are cultured on an elastic substrate which is mounted on a cell stretching device. The stretching device that reversibly deforms the elastic film. The stretcher itself is commonly mounted on an inverted microscope, where an objective underneath the elastic slide facilitates the imaging of dynamic cell behaviors upon stretching. To stretch the cells a prescribed strain is applied to the elastic substrate. **(B)** Microfluidic device. Consisting of a cell culture chamber with an inlet connecting to a syringe pump, and an outlet to collect the flow-through, the device can be used to study cellular responses to shear stress. Cells are seeded in the chamber. Medium is flown through at a prescribed rate. To collect images of cells over time, the device usually is mounted on an inverted microscope, where an objective is placed beneath the cell culture chamber.

Vascular cells are subjected to cyclic stretching due to the pulsating rhythm of the circulation and the respiratory cycle of inhalation and exhalation. Pulsatile blood flow coinciding with cardiac rhythms has been observed across blood vessels, including in capillary beds *in vivo* (PETERSON, 1954; Lee John et al., 1994; Santisakultarm et al., 2012)*.* Under pulsatile flow arteries dilate about 10% (Shimoda et al., 1998) and can alter the normal stress experienced by the blood vessel, though the significance of such alteration is yet to be studied. Changes in tensile forces or strain pertaining to such cyclic stretch can activate mechanosensitive protein and trigger a downstream biochemical signaling cascade. This process, known as mechanotransduction, can lead to both short- and long-term changes. To study the mechanotransduction upon stretching, many stretching devices have been developed. A common feature shared by the various devices is an elastic substrate (Figure 3) on which cells are cultured (Chen et al., 2013). This elastic substrate is subjected to reversible deformation through a variety of mechanisms, including vacuum chamber, mechanical stretch, or electromagnetic actuation (Moore et al., 1994; Naruse et al., 1998b; Kamble et al., 2017; Sato et al., 2019; Lien and Wang, 2021).

**FIGURE 4:**
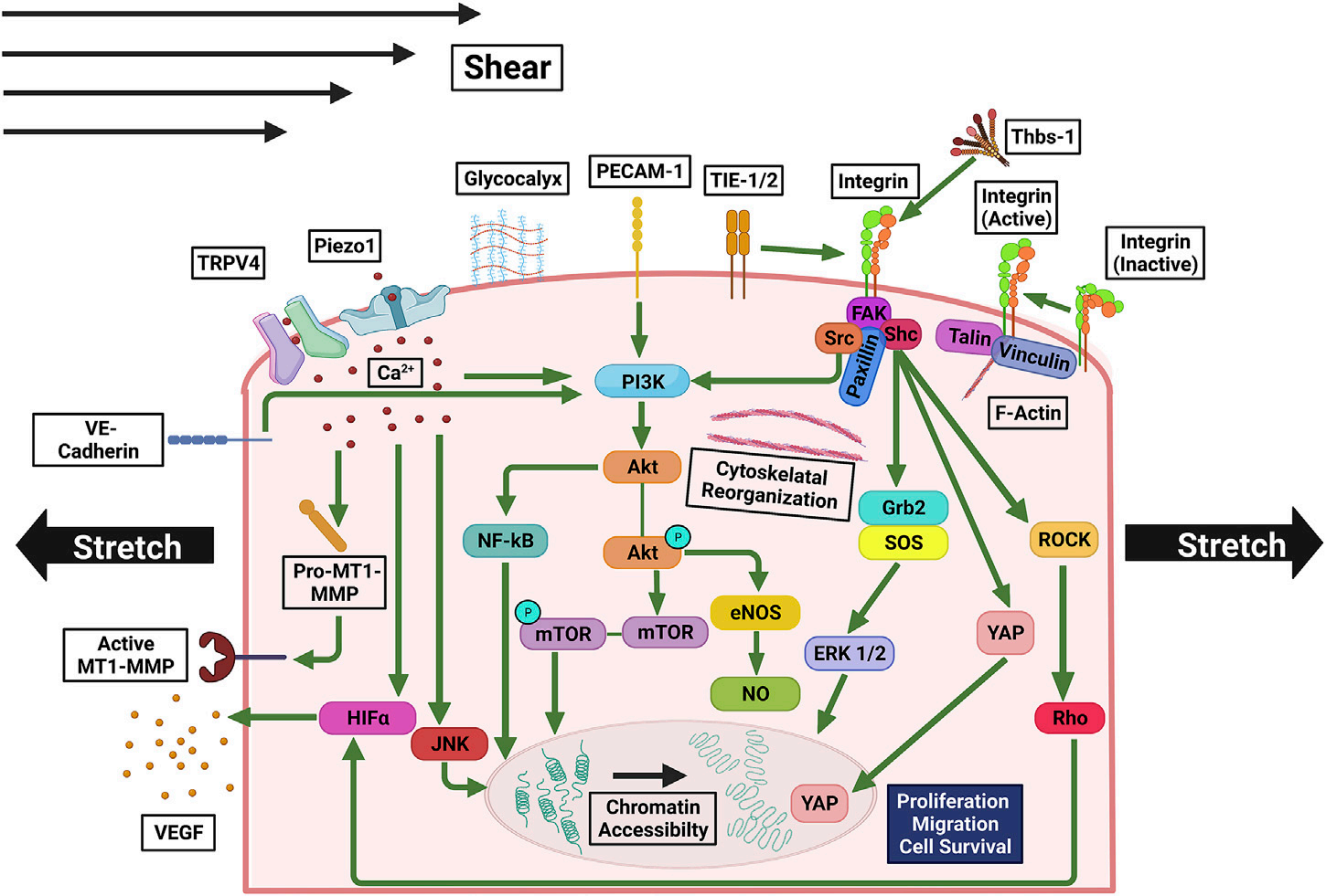
Mechanosensitive pathways involved in signaling triggered by stretching and/or shear stress. Ca^2+^ influx through Piezo1 and TRPV4 upregulates MT1-MMP, PI3K, JNK, promoting cell growth and proliferation, and HIFα, upregulating VEGF expression. PI3K is also upregulated by PECAM-1, integrin, and VE-Cadherin, which are activated by mechanical stress, and in turn, activate Akt. Akt’s downstream pathways activate NF-κB and mTOR, which facilitate cell growth and proliferation, as well as eNOS and NO, which enhance cell survival. Activated integrin, to which a macromolecular complex of FAK, Shc, Src, and Paxillin is bound, interacts with TIE-1/2. Rho and ROCK are activated by FAK and/or Src, upregulating in HIFα. Shc upregulates Grb2, SOS, and FRK 1/2, promoting cell proliferation and growth. Under shear stress and stretch YAP is translocated to the nucleus.

Immediately after stretching, the stretch-activated calcium channels, piezo type mechanosensitive ion channel component 1 (Piezo1), and transient receptor potential vanilloid-type 4 (TRPV4) open, allowing Ca^2+^ influx, which activates calpain (Figure 4), a Ca^2+^-dependent protease that facilitates pathological angiogenesis when in excess (Thodeti et al., 2009; Carmeliet and Jain, 2011; Nourse and Pathak, 2017). Moreover, Piezo1 activation upregulates membrane type 1-matrix metalloproteinase (MT1-MMP), Akt, and mTOR (Kang et al., 2019; De Felice and Alaimo, 2020). Genetic deletion and pharmacological inhibition of Piezo1 reduce endothelial sprouting and lumen formation. Downstream of the calcium signaling from Piezo1 and TRPV4 there is an increase in HIF-1α expression, as a result, VEGF expression is increased (Liu et al., 1995; Huang et al., 2021). Notably, HIF expression decreases after shear stress treatment, indicating that HIF activation induces stretch- but not shear-mediated angiogenesis (Milkiewicz et al., 2007). MAPK pathway (Liu et al., 2022) is also activated by Ca + influx. Downstream to Ca + influx stretch also transiently activates c-jun N-terminal kinase (JNK) and ERK in endothelial cells (Kaunas et al., 2006; Hsu et al., 2010), shifting the balance among physiological processes including proliferation, differentiation, apoptosis, and development (Wei and Liu, 2002).

In response to cyclic stretch smooth muscle cells release thrombospondin-1 (Thbs-1) (Yamashiro et al., 2020; Yamashiro and Yanagisawa, 2020). Thbs-1 binds to integrins to establish focal adhesion complexes leading to translocation of YAP to the nucleus (Yamashiro et al., 2020). Nuclear YAP promotes angiogenesis by serving as a transcriptional coregulator for genes involved in cytoskeletal dynamics, proliferation, and migration (Sheibani, 1999; Azad et al., 2019). Integrin activity can also be directly regulated by mechanical stretch. Upon stretch integrin changes to conformation to an active state (Figure 4) (Wilson et al., 1995; Askari et al., 2009; Ross et al., 2013). Integrin-mediated signaling in turn activates the activity of FAK, ERK1/2, Shc, growth factor receptor bound protein 2 (Grb2), protein kinase C (PKC), nuclear factor kappa B (NF-κB), protein kinase B (Akt), phosphoinositide 3-kinase (PI3K), which increases the sensitivity of the cells to growth factors (Zheng et al., 2004). Tyrosine kinases fetal liver kinase-1 (Flk-1), tyrosine kinase with immunoglobulin like and EGF like domains 1 (Tie1), and angiopoietin-1 receptor (Tie2) are also activated by stretch. During angiogenesis integrin and Flk-1 synergize their activities (Mahabeleshwar et al., 2007). Tie1 and Tie2 associate with integrin on the cell membrane (Dalton et al., 2016). Upregulation of these tyrosine kinases/receptors promotes cell survival, proliferation, migration (Wang et al., 2020), as well as actin polymerization, that contribute to angiogenesis (Mainiero et al., 1995; Brakebusch et al., 2002; Klein et al., 2002; Eapen et al., 2011; Zhao et al., 2016; Cooper and Giancotti, 2019; Romero et al., 2020). Moreover, integrin signaling also enhances the expression of PDGF, endothelin-1 (ET-1), and sterol regulatory element-binding protein-1 (SREBF1) (Meyer et al., 2000; Chen et al., 2001). Upon integrin activation, stress-induced sprout alignment occurs *via* the Rho-ROCK axis when endothelial cells elongate and reorient stress fibers perpendicular to the stretch direction (Yoshigi et al., 2003; Kaunas et al., 2005; Barron et al., 2007; Wilkins et al., 2015; Yang et al., 2017). If the Rho-ROCK axis is inactivated, endothelial cells exhibit delayed reorientation in response to stretch (Huang et al., 2012). The extent of stress fiber reorientation depends on the strain rate and is proportional to Rho activity (Kaunas et al., 2005).

Through the Rho-ROCK axis, stretch promotes endothelial sprouting and patterning (Wilkins et al., 2015). Cyclic stretch results in endothelial sprouting by upregulating PDGF (Yu et al., 2009) by Src-tyrosine kinase and Rho pathways (Naruse et al., 1998a; Kimura and Eguchi, 2009; Yu et al., 2009). PDGF secreted by endothelial cells recruits perivascular cells attached to the blood vessel wall (Hellstrom et al., 1999). Stretch also promotes cell migration. Cells cyclically stretched with a strain rate of 13% migrate 15-fold faster than the static control because of Rho and MAPK activation (Huang et al., 2004; Kimura and Eguchi, 2009; Yu et al., 2009; Murphy et al., 2015; Ridley, 2015). Cyclic stretch alters the integrin receptor subpopulations in endothelial cells, where intercellular adhesion molecule-1 (ICAM-1) increases and vascular cell adhesion molecule-1 (VCAM-1) decreases (Barron et al., 2007). ICAM-1 is known to remodel the cytoskeleton and thus modulate cell migration (Kevil et al., 2004; Yang et al., 2006). Furthermore, cyclic stretch promotes angiogenic sprouting and aligns new sprouts perpendicular to the direction of stretch by receptor tyrosine kinase and Rho pathways (Wilkins et al., 2015).

VE-cadherin converts mechanical stretch to proliferative signals (Figure 4) (Liu et al., 2007). Downstream of VE-cadherin Akt signaling is initiated which induces cell proliferation in response to strain (Li and Sumpio, 2005; Gavard and Gutkind, 2008). Akt signaling also inhibits apoptosis in response to stretch (Liu et al., 2003). Furthermore, as a response to calcium influx, the endothelial nitric oxide synthase (eNOS) is activated (Barbeau et al., 2021). Nitric oxide (NO) production and the expression of eNOS increased during the cyclic stretch of endothelial cells (Takeda et al., 2006). Along with vasodilation, NO signaling contributes to cell survival and cell-growth proliferation, migration, differentiation in endothelial progenitor cells, and survival (Cooke and Losordo, 2002; Lähteenvuo and Rosenzweig, 2012).

Furthermore, stretch can promote angiogenesis by chromatin reorganization (Figure 4). Cell stretching results in a change in chromatin architecture which promotes chromatin accessibility (Nava et al., 2020; Zhang et al., 2021). The changes to chromatin can directly influence transcription (Nava et al., 2020). It is possible that stretch directly upregulates pro-angiogenic gene expression by softening heterochromatin and exposing originally hidden promoters. Further investigation can elucidate the specific gene expression profiles. Stretch regulates differentiation, proliferation, and survival pathways in endothelial cells.

### Shear

On the luminal side of blood vessels, endothelial cells are exposed to shear stress due to blood flow. There is a shear stress threshold and when this threshold is surpassed sprouting angiogenesis occurs (Galie et al., 2014). This provides a mechanism for fluid flow to mold vasculature. Shear stress also regulates the endothelial barrier function. Shear stress aligns endothelial cells and under higher shear stress the permeability of the endothelial cells decreases (Buchanan et al., 2014). Elevated shear stress also results in endothelial cell proliferation (Egginton et al., 2001). To model shear stress cells are cultured in flow chambers (Galie et al., 2014; Russo et al., 2020), through which medium or buffer is flown at a prescribed rate (Figure 3).

Mechanosensors of shear stress include ion channels Piezo1 and TRPV4, tyrosine kinase receptors (TKRs), G-protein coupled receptors (GPCRs), integrins, intercellular junction molecules, VE-cadherin and platelet endothelial cell adhesion molecule-1 (PECAM-1), and the glycocalyx (Figure 4) (Tzima et al., 2001; Jong Lee and Young Koh, 2003; Fleming et al., 2005; Chachisvilis et al., 2006; Conway et al., 2013; Ranade et al., 2014; Zeng and Tarbell, 2014; Zhou et al., 2014; Swain and Liddle, 2021). Following shear stress calcium channels such as Piezo1 and TRPV4 are activated (Mendoza et al., 2010; Ranade et al., 2014). The TKR Tie1 is suppressed while Tie2 is activated (Chen-Konak et al., 2003; Jong Lee and Young Koh, 2003). Shear stress mediates mechanotransduction *via* integrins by inducing a conformation change to an active state (Tzima et al., 2001). In intercellular junctions, there is a decrease in tension across VE-cadherin and an increase in tension across PECAM-1 (Conway et al., 2013). The glycocalyx is the endothelial surface layer consisting of glycoproteins, proteoglycans, and glycosaminoglycans. The glycocalyx is located on the apical side of endothelial cells and serves as an interface between blood flow and the cell membrane (Pahakis et al., 2007). High shear stress disrupts the endothelial luminal glycocalyx (Brown et al., 1996). Following exposure to shear stress the glycocalyx is remodeled (Zeng and Tarbell, 2014).

Shear-induced calcium signaling contributes to angiogenesis by activation of PI3K/Akt and eNOS (Figure 4) (Wang et al., 2016). In endothelial progenitor cells, eNOS signaling contributes to endothelial cell differentiation, proliferation, and tube formation (Lu et al., 2015). Tie2 also activates Akt and eNOS (Yang et al., 2012). In cells treated with a pan-NOS inhibitor, the rate of endothelial migration becomes insensitive to shear stress (Song and Munn, 2011). Another way Tie2 contributes to angiogenesis is by activating the MAPK pathway *via* Grb2 (Huang et al., 1995). Upon shear-activation, integrins in turn activate Rho (Tzima et al., 2001; Urbich et al., 2002), which activates MAPK resulting in cell growth and proliferation (Vojtek and Cooper, 1995). Through cytoskeleton remodeling, Rho also promotes cell migration and cellular alignment to flow. Moderate shear stress exerted by interstitial flow suffices to induce endothelial morphogenesis and sprouting (Song and Munn, 2011). Under shear stress VE-Cadherin binds to VEGFR2 and VEGFR3, leading to ligand independent PI3K/Akt signaling (Coon et al., 2015; Wang et al., 2020). PECAM-1 activates PI3K/Akt signaling as well, along with the eNOS pathway for NO production (Fleming et al., 2005) and subsequent glycocalyx remodeling-dependent angiogenesis. Moreover, shear stress influences cellular behavior by changes in gene expression. Endothelial genes transiently upregulated by shear stress include c-FOS, PDGFA, PDGFB, EGR1, ICAM1, and MCP1. Endothelial genes with sustained upregulation in response to shear stress are NOS3, COX2, tPA, Smad6, Smad7, MADH6, MADH7, SOD, and TGF-β (Topper and Gimbrone, 1999). Endothelial genes downregulated in response to shear stress are Endothelin and VCAM1 (Topper and Gimbrone, 1999). During exposure to shear stress mechanoreceptor activation of the Rho, MAPK, and Akt pathways along with shear stress mediated chromatin remodeling lead to changes in gene expression (Dimmeler et al., 1998; Jalali et al., 1998; Illi et al., 2003; Shiu et al., 2004; Tsaryk et al., 2022). We note in addition to the fluid shear forces and stretch forces in the tangential direction, endothelial cells are subjected to normal forces in capillaries, imposed by hydrostatic pressure of blood in the circulatory system (Bazmara et al., 2015). Based on the model, such pressure can be as high as 38 dyne/cm^2^ in the capillary (Akbari et al., 2018), higher than the pressure caused by fluidic shear (Stapor et al., 2011). It was reported the normal force can suppress proliferation in endothelial cells, though the detailed molecular mechanism is still under investigation (Akbari et al., 2019). Despite the intriguing finding, we note the estimation of normal stress is based on measurements made in microfluidic devices, not *in vivo* and the result was not widely validated by other groups. Moreover, the mechanism by which normal forces regulate angiogenesis is not clear.

While stretch and shear stress both activate angiogenesis through many of the same mechanotransducers, there are differential effects between the two. For example, shear stress leads to peripheral accumulation of focal adhesions, while stretch leads to randomly distributed focal adhesions (Shikata et al., 2005). Shear stress also increases the transendothelial electrical resistance while stretch does not (Shikata et al., 2005). Shear stress predominantly increases total superoxide production, fibronectin expression, and gelatinase activation; however, stretch reduces the expression of these in endothelial cells (Thacher et al., 2009). Other differences are that stress inactivates angiostatin II receptor 1 (AT1R) through a NO-dependent pathway while stretch activates AT1R in a ligand dependent manner (Lu and Kassab, 2011). MMP-2 levels are increased by stretch, but not shear stress stimulation (Rivilis et al., 2002). Given that endothelium in the blood vessels experience both shear and stretch forces, it is likely that these forces work synergistically, enhancing certain mechanosignaling events, and sometimes work antagonistically, keeping certain signaling events in check, during the formation of new blood vessels (Zhao et al., 1995).

## *In vitro* model systems to study mechanical regulators of angiogenesis

To study the mechanical effects on angiogenesis longitudinally and *via* long-term imaging, *in vitro* model systems are often used, where mechanical parameters (e.g., force, strain rate, shear rate, stiffness, etc.) can be precisely controlled, and sometimes dynamically changed. An essential component of model systems for angiogenesis is endothelial cells. Commonly used endothelial cells include bovine aortic endothelial cells (BAECs), human umbilical vein endothelial cells (HUVECs), and human microvascular endothelial cells (HMEC-1s) (Table 1). BAECs are primary cells derived from cows (Schwartz, 1978), thus representing human endothelial cells to a lesser degree. HUVECS are routinely harvested in large numbers from medical waste (Hauser et al., 2017; Kocherova et al., 2019), though these cells cannot be perpetuated more than 5–10 passages (Liao et al., 2014). HMEC-1 is an immortalized human microvascular cell line that can be passed at a higher number (Ades et al., 1992) without showing signs of senescence, making these cells more suitable for longer-term studies (Ades et al., 1992).

**TABLE 1:** Available tools for *in vitro* evaluation of the mechanical regulation of anagenesis.


Cell line	BAEC (Sato et al., 1987; Sumpio et al., 1987; Letsou et al., 1990; Malek and Izumo, 1992; Awolesi et al., 1995; Jacobs et al., 1995; Hishikawa and Lüscher, 1997; Kataoka et al., 1998; Kohn et al., 2015; Mahmoud et al., 2021)
	HUVEC (Illi et al., 2003; Zheng et al., 2004; Yu et al., 2009; Dickinson et al., 2012; Jalali et al., 2015; Zhao et al., 2018; Tsaryk et al., 2022)
	HMEC-1 (Ades et al., 1992; Wilson et al., 2009; Groothuis et al., 2010; Xie et al., 2020; Katari et al., 2021)
Material	Collagen (Krishnan et al., 2008; Lee et al., 2013; Mason et al., 2013; Wilkins et al., 2015)
	Matrigel (Ades et al., 1992; Mammoto et al., 2009; Wilson et al., 2009; Yan et al., 2020)
	Fibrin (Groothuis et al., 2010; Morin and Tranquillo, 2013; Wilkins et al., 2015)
	PDMS (Dickinson et al., 2012; Rosenfeld et al., 2016; Sivarapatna et al., 2017; Zhao et al., 2018; Zeinali et al., 2021)
	Polyacrylamide gel (Deroanne et al., 2001; Saunders and Hammer, 2010; Jalali et al., 2015; Santos et al., 2015; Mahmoud et al., 2021)
Mechanical Force	Geometry (Micropatterning) (Chen et al., 1997; Dike et al., 1999; Liu et al., 2007; Lei et al., 2012)
	Stretch (Cell Stretcher) (Malek and Izumo, 1992; Awolesi et al., 1995; Barron et al., 2007; Tremblay et al., 2014; Loerakker et al., 2018)
	Shear (Microfluidic Chamber) (Song and Munn, 2011; Buchanan et al., 2014; Galie et al., 2014; Akbari et al., 2018; Kuzmic et al., 2019)

To study the effects of stiffness, cells are either cultured on top or within ECM, such as collagen, Matrigel, and fibrin, or ECM-coated substrates, such as alginate, PDMS, polyacrylamide, and polyethylene glycol (Caliari and Burdick, 2016). The elastic moduli of these substrates can be controlled by the density of the polymer or the extent of inter-polymer crosslinking (Caliari and Burdick, 2016; Zhao et al., 2018; Guo et al., 2022). To study the effect of ligand availability and curvature, endothelial cells are seeded on micro-patterns to form islands, lines, half-channels, or channels so that the phenotypes and the behaviors across various geometries can be compared, and the key geometric characteristic contributing to endothelial sprouting can be identified (Figure 2) (Dike et al., 1999; Mandrycky et al., 2020). To interrogate how different degrees of stretch affects angiogenesis, cells are first seeded on an elastic film, often with a compatible refractive index to facilitate imaging-based observations (Yu et al., 2009; Yan et al., 2020; Lien and Wang, 2021). A prescribed strain will then be applied to the elastic film (Figure 3), through a motorized stepper, a vacuum, or other stretching mechanisms. The dynamic changes in stretched cells, such as shape, cell-substrate adhesion, ion fluxes, and enzymatic activities, can be monitored through timelapse live cell imaging. We note that *via* pulsating rhythm of the circulation and the respiratory cycle of inhalation and exhalation, cells are stretched *in vivo* both circumferentially and axially. Therefore, the endothelium lining the blood vessels may be treated as warped 2D sheets of cells. To recapitulate the physiological stretching conditions a biaxial stretching system is preferred (Tremblay et al., 2014; Imsirovic et al., 2015; Shiwarski et al., 2020). To model the effect of shear stress, microfluidic devices are often used, which consist of a cell culture chamber and tubing connecting the chamber to a fluid pump (Buchanan et al., 2014; Galie et al., 2014). The fluids can be flown through the chamber at prescribed rates, applying shear stress and emulating hemodynamic conditions *in vivo* (Park et al., 2017, 2019). The cell culture chamber is typically designed so that cells can be imaged by microscopy under flow conditions (Figure 3).

Moving forward, tissue engineering approaches, such as organ-on-chip microfluidics, micro-engineered 3D scaffolds, and bioprinting are of high interest because these systems provide on-demand control of the geometry, substrate stiffness, matrix composition, cells with selective gene expression, as well as external mechanical force application. Moreover, incorporating human endothelial cells in these models may provide insights that are specific to human angiogenesis. Bioprinting is an emerging fabrication method for bioengineered tissues. Bioprinting is the use of biomaterials such as ECM and 3D printing technology to fabricate tissue constructs mimetic of native tissue organizations. Due to its reproducibility and rapid production, the employment of bioprinting in the study of angiogenesis is on the rise.

It has been shown that functional vasculatures can be formed using bioprinting. In one example a cell-gelatin mixture was printed inside the collagen layer. The gelatin served as a sacrificial template and was liquefied. The ends of the channel were connected to a pump and the medium was perfused through the system. Within 5 days an endothelial channel was formed (Lee et al., 2014). In another study, vessels were formed by printing using coaxial nozzles. In this study, endothelial cells were encapsulated on an ECM/alginate bioink, which served as the shell. A sacrificial material, Pluronic F-127, served as the core. The vessels were printed in a flexible pattern and perfused. The formation of an intact endothelium was observed by day 7 (Gao et al., 2018). Techniques like freeform reversible embedding of suspended hydrogels allow low viscosity bioinks to be printed in a support bath to create blood vessels (Kreimendahl et al., 2021). In addition to extrusion-based bioprinting, vessels can also be formed using stereolithography bioprinting. In stereolithography, the structure is cured layer by layer (Huang et al., 2020). Cell-laden hydrogels have been printed using visible light stereolithography to form vessel structures (Elomaa et al., 2015; Grigoryan et al., 2019). Vasculature bioprinting also can be combined with other forms of tissue engineering, to vascularize tissue constructs or organoids (Pitaktong et al., 2020). Without gas and nutrient/waste exchange facilitated by the biofabricated vasculature, engineered tissues are limited in growth and may develop necrotic cores in their inner parts. It was shown that bioprinted, vascularized tissues exhibit angiogenesis and support *in vivo* integration of implanted engineered tissue (Maiullari et al., 2018). Beyond constructing hollow, tubular structures for blood vessel formation, bioprinting can also be used to define patterns of growth factor release (Freeman et al., 2020). Both spatial and temporal release can be controlled. As tissue engineering strategies advance better models of angiogenesis will be developed.

## Future directions

Mechanical and biochemical pathways act in concert to promote sprouting angiogenesis through several pathways involving cell migration, proliferation, and survival. The mechanochemical regulation of angiogenesis is a relatively new topic, and additional mechanotransducers and mechanotransduction pathways will likely be uncovered. For example, factors such as extracellular fluid viscosity, osmotic pressure, hydrostatic pressure, and compression can also alter the course of angiogenesis, but the detailed molecular mechanisms beyond such alterations are yet to be determined (Park et al., 2020). Recent evidence has shown that elevated extracellular viscosity enhances cell motility (Pittman et al., 2022), which may contribute to angiogenesis in regions of higher blood viscosity like the tumor microenvironment. Tissue engineered-based *in vitro* models capable of multiplexing are highly desirable so that multiple mechanical factors can be applied at varying quantities simultaneously. Monitoring bioprinted blood vessels can help discern which signaling pathways are enhanced synergistically by stretch and shearing, which are regulated additively, and which are regulated antagonistically.

Both mechanical and biochemical pathways of angiogenesis are co-opted by life-threatening diseases, including cancer or atherosclerosis. Studying mechanical regulation of angiogenesis may inspire novel strategies to reverse pathological angiogenesis mechanically. For example, by relaxing the stretching stress can angiogenesis be halted or suppressed? Moreover, certain mechanical treatment might be applied along with drugs treating angiogenesis, to improve the anti-angiogenesis efficacy. Anti-angiogenic drugs often fail to effectively block angiogenesis because of the redundancy of angiogenic pathways. This is especially true in tumor progression. When one pathway is blocked the tumor may induce angiogenesis with a different pathway while developing resistance against the antiangiogenic drug. Indeed, studies by the Jain group have hinted that by lowering interstitial fluid pressure in the tumor microenvironment, the leaky vessels with angiogenic sprouting start to trim and regain the barrier (Jain, 2013). Developing mechanical treatments to aid in the suppression of tumor angiogenesis is worth exploring.

Recent advances in spatial mapping of single-cell -omics make it possible to study single-cell level genomics, epigenomics, and proteomics of endothelial cells while subjecting them to mechanical perturbations known to alter angiogenesis. This will lead to greater insight into the downstream signaling of mechanotransducers, and the discovery of new mechanotransducers in the cell.
